# Orthopaedic Aspects of Marfan Syndrome: The Experience of a Referral Center for Diagnosis of Rare Diseases

**DOI:** 10.1155/2016/8275391

**Published:** 2016-12-05

**Authors:** Fernando De Maio, Alessandro Fichera, Vincenzo De Luna, Federico Mancini, Roberto Caterini

**Affiliations:** Department of Orthopaedics and Traumatology, University of Rome “Tor Vergata”, Viale Oxford 81, 00133 Rome, Italy

## Abstract

Marfan syndrome is caused by mutations in the fibrillin-1 gene (*FBN1*). The most important features affect the cardiovascular system, eyes, and skeleton. The aim of this study was to report the most frequent musculoskeletal alterations observed in 146 patients affected by Marfan syndrome. Fifty-four patients (37%) underwent cardiac surgery and 11 of them received emergent surgery for acute aortic dissection. Ectopia lentis was found in 68 patients (47%) whereas myopia above 3D occurred in 46 patients (32%). Musculoskeletal anomalies were observed in all patients with Marfan syndrome. In 88 patients (60.2%), the associated “wrist and thumb sign” was present; in 58 patients (39.7%), pectus carinatum deformity; in 44 patients (30.1%), pectus excavatum; in 49 patients (33.5%), severe flatfoot; in 31 patients (21.2%), hindfoot deformity; in 54 patients (36.9%), reduced US/LS ratio or increased arm span-height ratio; in 37 patients (25.3%), scoliosis or thoracolumbar kyphosis; in 22 patients (15%), reduced elbow extension (170° or less). Acetabular protrusion was ascertained on radiographs in 27 patients (18.4%). Orthopaedic aspects of the disease are very important for an early diagnosis; however, we have not observed definite correlations between the extent of orthopaedic involvement and aortic complications.

## 1. Introduction

Marfan syndrome (MFS) is a variable autosomal dominant disorder of the connective tissue caused by mutations in the fibrillin-1 gene on chromosome 15 encoding the microfibrillar protein fibrillin-1 [1].

This disease of connective tissue occurs worldwide and affects both sexes equally. Its prevalence has been estimated at 2-3 persons per 10,000 [2]. About 25%–30% of the cases of Marfan syndrome represent sporadic mutations. The phenotypic features of Marfan syndrome are tall stature, arachnodactyly, disproportionately long and thin limbs, skin striae, and joint laxity. The disease involves several body systems but the most important features affect the cardiovascular system, eyes, and skeleton. The diagnosis of Marfan syndrome is defined by the Ghent criteria [3]. In 2010, Loeys et al. [4] published revised Ghent criteria for the Marfan syndrome. A comparative analysis on different retrospective data sets has shown about 90% correlation between the original and revised Ghent criteria. For the diagnosis of Marfan syndrome, it is often necessary to have collaboration among ophthalmologists, paediatricians, cardiologists, cardiac surgeons, orthopaedic surgeons, and geneticists. With regard to the orthopaedic features in the new Ghent criteria of 2010, much importance, compared to the old Ghent nosology of 1996, is given to the combined wrist and thumb sign, acetabular protrusion, hindfoot deformity, and pectus carinatum. Inability to detect a mutation in* FBN1* or a molecular abnormality in fibrillin-1 does not exclude the diagnosis of Marfan syndrome in a person who meets the clinical criteria. In effect, mutation in fibrillin-1 on chromosome 15 is detected in 66%–91% of cases [5]. The more severe clinical features of this disorder are represented by the aortic root aneurysm and ectopia lentis. However, musculoskeletal involvement in Marfan syndrome, even if less drastic, is often more evident than other pathological features and for this reason the orthopaedic aspects of the disease are fundamental for a suspicious diagnosis of this pathological condition. In fact, in the first reported case, Antoine-Bernard Marfan described 5-year-old Gabrielle with skeletal manifestations of the disease [6].

The most important musculoskeletal abnormalities in patients with Marfan syndrome are “wrist sign,” “thumb sign,” pectus carinatum deformity, pectus excavatum or chest asymmetry, hindfoot deformity, severe flatfoot, dural ectasia, protrusio acetabuli, reduced upper segment/lower segment (US/LS) and increased arm span-height ratio, scoliosis, kyphosis, and reduced elbow extension.

Medial protrusion of the femoral head (protrusio acetabuli) is common in Marfan syndrome [7, 8]. Prolonged acetabular protrusion may result in secondary osteoarthritic changes in the hip joint [8, 9].

Dural ectasia is an enlargement of the outer layer of the meningeal sac. It is very common in Marfan syndrome [10, 11] and it is a specific major criterion in the Ghent diagnostic classification [3]. In the majority of cases, dural ectasia is not associated with back pain but the pathological condition may be discovered by CT and/or MRI of the spine [12]. However, CT or MRI of the spine was not performed during our screening due to the high cost of the exam and we evaluated this data only in the few cases who had practiced MRI independently.

In the present paper, we report the orthopaedic aspects of 146 patients affected by Marfan syndrome examined at the Marfan Presidium of the Tor Vergata University Hospital of Rome (Italy) which is a referral center for the diagnosis of rare diseases.

## 2. Materials and Methods

In the last five years, 500 patients were screened at the Marfan Presidium of the Tor Vergata University Hospital of Rome (Italy). In 60 patients, the diagnosis of MFS was already made before our screening but they had chosen to be followed up at our presidium which is a referral center for the diagnosis of rare diseases. The other patients who were not diagnosed with MFS yet came to our center for suspected MFS as referred by their family doctor, paediatrician, or cardiologist or by a self-made diagnosis based on Internet readings.

The diagnosis of Marfan syndrome until 2010 has been made according to the Ghent criteria [3] and subsequently according to the revised Ghent criteria [4]. The diagnosis of Marfan syndrome has been made using a multidisciplinary approach. All patients were evaluated by a team that included a cardiac surgeon, a cardiologist, an orthopaedic surgeon, an ophthalmologist, an odontologist, and a paediatrician. Paediatric counselling was given only in patients under 14 years of age. All the patients received genetic counselling but genetic sampling was performed only in case of uncertain diagnosis while it was avoided when the clinical diagnosis was striking or already made previously due to the high cost of the test. Standing AP and LL X-rays of the spine, including the hips to evaluate the depth of the acetabulum, were done in all patients. Protrusio acetabuli is clinically characterized by hip joint stiffness and pain. An important radiographic finding is represented by an increased center-edge angle of Wiberg [13]. A vertical line drawn through the center of the femoral head and a line from the femoral head and the center to the upper outer margin of the acetabulum form the center-edge angle of Wiberg. A center-edge angle of 20°–40° is considered normal for adults and an angle over 40° indicates acetabular protrusion. Orthopaedic examination was performed to look for the typical skeletal manifestations of the Marfan syndrome. Particular attention was paid to discover the presence of “wrist sign” [14] and “thumb sign” [15], pectus carinatum deformity, pectus excavatum or chest asymmetry, hindfoot valgus in combination with forefoot abduction and lowering of the midfoot, acetabular protrusion, reduced upper segment to lower segment (US/LS) ratio (for white adults < 0.85) and increased arm span-to-height ratio (for adults > 1.05), scoliosis or thoracolumbar kyphosis, and reduced elbow extension.

The “thumb sign” is positive when the thumb extends well beyond the ulnar border of the hand when overlapped by the fingers. The “wrist sign” is positive when the thumb overlaps the fifth finger when grasping the contralateral wrist. Elbow extension is considered reduced if the angle between the arm and forearm measures 170° or less upon full elbow extension.

MRI of the lumbosacral spine was evaluated to detect the presence of dural ectasia, which represents one of the major diagnostic criteria for the Marfan syndrome, only in the patients who had practiced this exam independently since CT or MRI of the lumbosacral spine was not included in our protocol.

## 3. Results

Marfan syndrome was confirmed in 146 patients (29.2%, 76 females and 70 males). At first, 28 patients (19%), ≤ 14 years old, were observed; 18 (12%) were 15–19 years old; 64 (44%) were 20–40 years old; and 36 (25%) were >40 years old. In all 354 patients who were not affected by MFS, skeletal abnormalities were more or less present, and therefore they continued to be followed up in our department of orthopaedics only for such problems. In 225 patients, deformities of the chest and/or of the spine were predominantly present, while in 129 patients deformities of the upper or lower limbs were predominantly present. In 86 patients (59%), the diagnosis of Marfan syndrome was made at Marfan Presidium of the Tor Vergata University Hospital in Rome. In 60 patients (41%), the diagnosis of Marfan syndrome had already been done in another hospital. In the 86 patients who were diagnosed with Marfan syndrome at our presidium, 38 patients were diagnosed according to the Ghent criteria [3]. 43 patients were diagnosed with Marfan syndrome according to the revised Ghent criteria [4]. In 5 patients, observed between 2008 and 2010 and suspected to be affected by Marfan syndrome, the final diagnosis was confirmed according to the revised Ghent criteria [4]. In 89 patients (61%), family history for Marfan syndrome was positive.

In 75 patients (51%), the geneticist decided to conduct genetic sampling; however, we only have the final results of the molecular test for* FBN1* mutation in 31 patients. 30 of these patients showed fibrillin-1 mutation. Surprisingly, the only patient who did not show a fibrillin-1 mutation suffered from a dissecting aneurysm of the aorta. No genes other than* FBN1* were tested.

Fifty-four patients (37%) have had cardiac surgery on the aortic root or on the mitral valve or had combined surgery. Twenty-eight patients (52%) were operated on at our hospital whereas 26 patients had had surgery before our screening. In general, 11 of the 54 patients operated on received emergent surgery for acute aortic dissection. All patients who underwent cardiac surgery presented obvious skeletal deformities. Such deformities, however, were present, more or less, also in the 92 patients who did not have cardiac surgery and therefore we did not observe a secure statistical correlation between the extent of orthopaedic involvement and aortic complications.

Sixty-eight patients (47%) had ectopia lentis that was classified as subluxated in 39 cases and luxated in 29 cases. Myopia above 3D occurred in 46 patients (32%).

Skeletal anomalies were observed in all 146 patients with Marfan syndrome.

**Figure 1:**
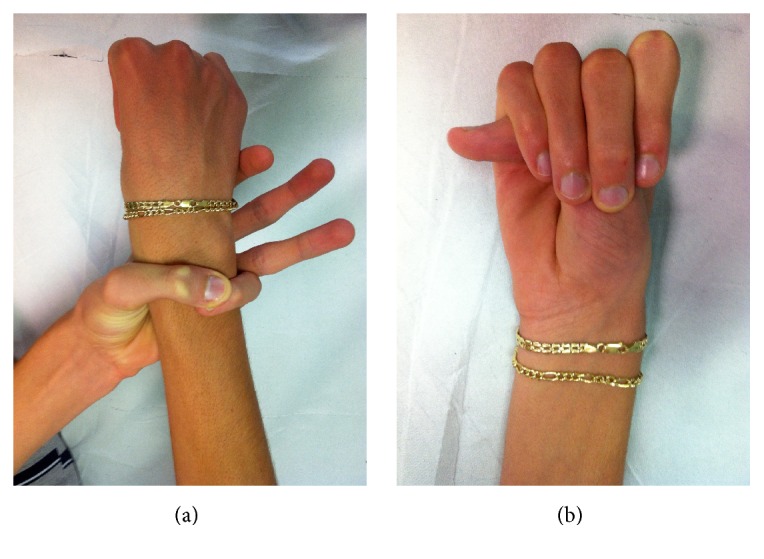
A twenty-year-old female affected by Marfan syndrome with an associated wrist and thumb sign. The “wrist sign” is positive when the thumb overlaps the fifth finger when grasping the contralateral wrist. The “thumb sign” is positive when the thumb extends well beyond the ulnar border of the hand when overlapped by the fingers.

**Figure 2:**
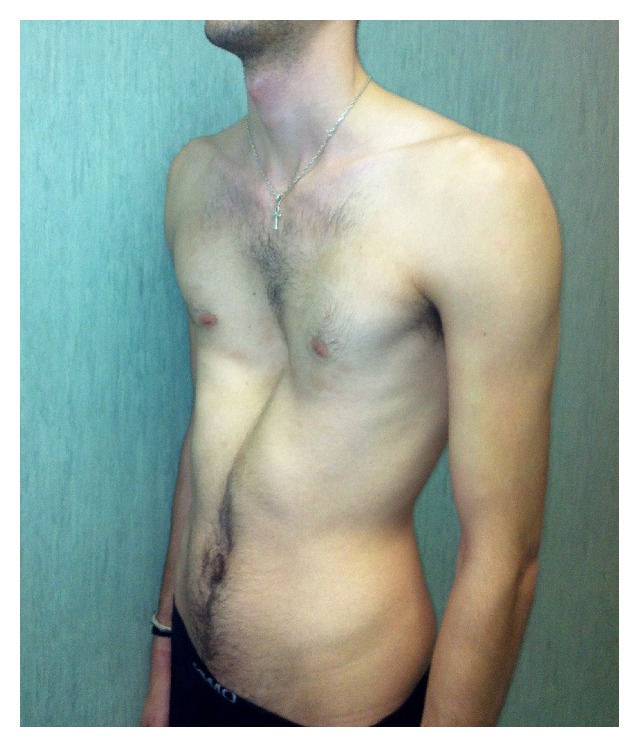
Severe pectus excavatum in a 29-year-old man affected by Marfan syndrome. The patient did not complain of any respiratory problem.

**Figure 3:**
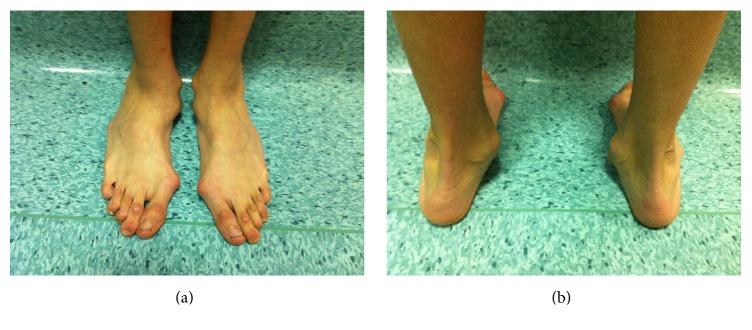
Hindfoot deformity with a marked valgus heel in a 23-year-old man affected by Marfan syndrome.

In 88 patients (60.2%), the associated wrist and thumb sign (Figure 1) was present; the isolated wrist or thumb sign was present in 6 patients (4.1%); pectus carinatum deformity was present in 58 patients (39.7%) and pectus excavatum or chest asymmetry was observed in 44 patients (Figure 2); hindfoot deformity was present in 31 patients (21.2%) (Figure 3); and severe flatfoot was present in 49 patients (33.5%).

**Figure 4:**
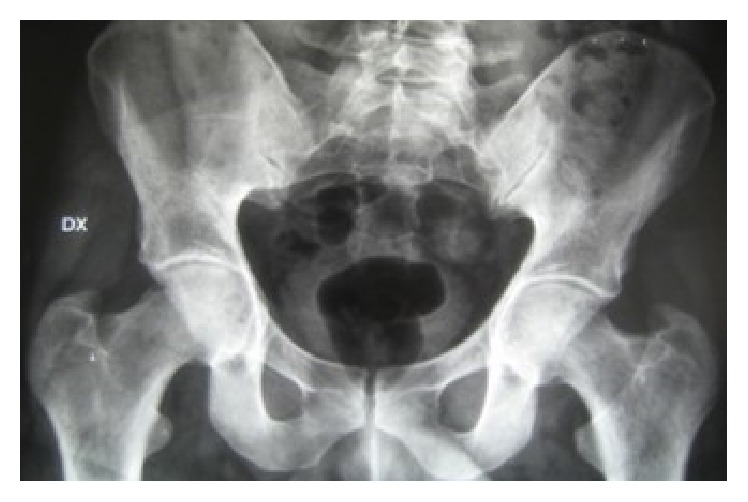
AP X-ray of the pelvis detects acetabular protrusion in a 36-year-old patient affected by Marfan syndrome. The patient complained of hip pain and clinical examination showed a restricted range of motion of the hips.

Acetabular protrusion was ascertained on radiographs in 27 patients (28.4%) (Figure 4), measuring the center-edge angle of Wiberg. All of these patients had hip pain and a limited rotation and abduction of the hip joint. In most patients, these symptoms were not severe, but in three cases we observed clinical and radiographic signs of severe osteoarthritis. In the remaining 119 patients (81.6%) with MFS, the radiographs of the pelvis did not show acetabular protrusion.

**Figure 5:**
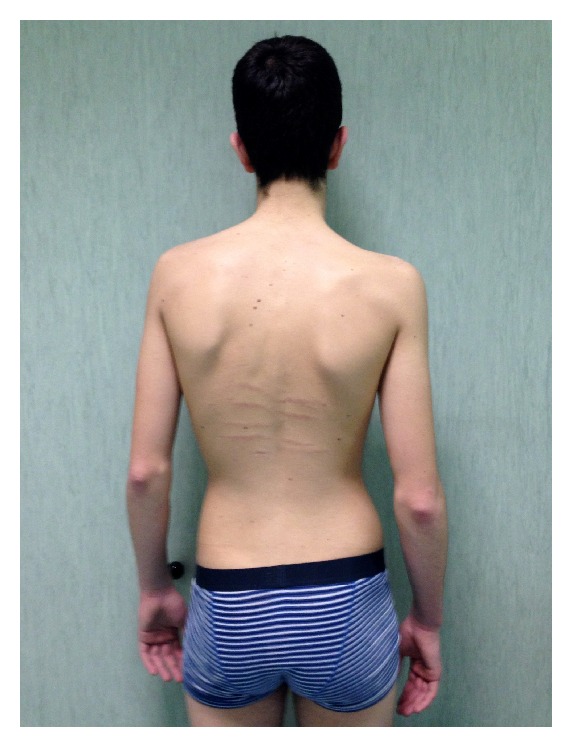
Twenty-five-year-old man affected by Marfan syndrome with scoliosis (>20°) in addition to dorsal skin striae and reduced elbow extension (<170°).

Reduced upper segment to lower segment (US/LS) ratio or increased arm span-to-height ratio was present in 54 patients (36,9%). At standing AP and LL X-rays of the spine, scoliosis > 20° or thoracolumbar kyphosis, measured by Cobb method, was observed in 37 patients (25.3%). Reduced elbow extension (170° or less) was measured in 22 patients (15%) (Figure 5). In 3 patients (2%), MRI of the lumbar spine showed dural ectasia. None of these patients had back pain or headaches or presented neurologic deficits.

In our research, the diagnosis of Marfan syndrome was made in 28 children under 14 years of age. The average age at diagnosis was 10.2 years (range: 4 y 10 m–13 y 10 m). In all of these children, we observed skeletal anomalies. In 18 children (64.2%), the associated wrist and thumb sign was present; the isolated wrist or thumb sign was observed in only 1 child (3.5%); pectus carinatum deformity was present in 9 children (32.1%); pectus excavatum or chest asymmetry was observed in 8 children (28.5%); hindfoot deformity was present in 13 children (46.4%); severe flatfoot was observed in 17 children (60.7%). Acetabular protrusion was ascertained on radiographs in 4 children (14.2%); reduced upper segment to lower segment (US/LS) ratio and/or increased arm span-to-height ratio was observed in 11 children (39.2%). At standing AP and LL X-rays of the spine, scoliosis > 20°, measured by Cobb method, or thoracolumbar kyphosis was observed in 5 children (17.8%). Reduced elbow extension (170° or less) was measured in 3 children (10.7%). No children had MRI of the lumbar spine to diagnose dural ectasia.

**Figure 6:**
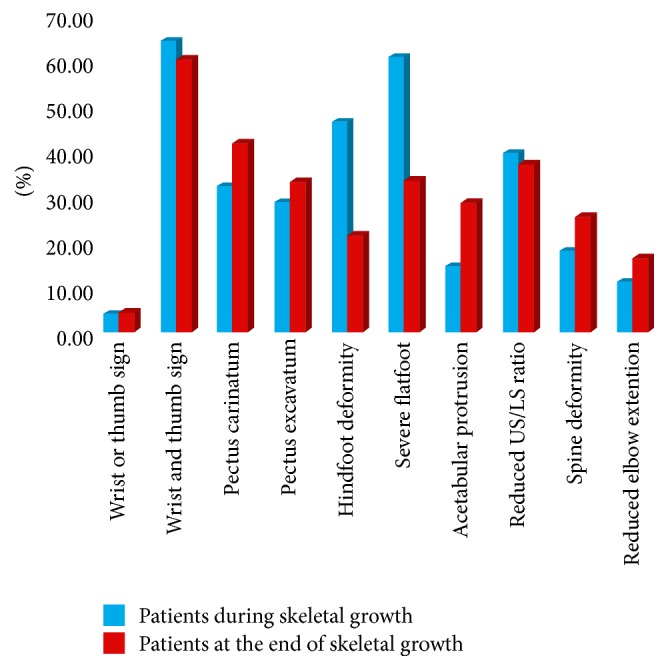
The histogram shows the prevalence of skeletal deformities in 146 patients affected by Marfan syndrome before and after the end of skeletal growth.

As summarized in the histogram (Figure 6) that shows skeletal deformities in our cohort of patients, the deformities of the limbs are prevalent in children.

## 4. Discussion

Marfan syndrome is a variable autosomal dominant disease caused by mutations in the fibrillin-1 gene on chromosome 15 [1, 5]. Although the pathogenesis of this syndrome is correlated to a defective gene, the diagnosis is based on clinical criteria and on family history. Since 1996, the diagnosis of Marfan syndrome has been made using Ghent criteria [3] and, more recently, by revised Ghent criteria [4]. In spite of the classic or revised Ghent criteria, diagnosis of Marfan syndrome remains complex but an early diagnosis is fundamental to identify patients at risk for acute aortic events and to improve the prognosis and lifestyle of these patients. Marfan syndrome, in fact, is known to be one of the highest risk conditions for aortic dissection and, as mentioned in the revised Ghent criteria [4], “it is important to avoid misdiagnosis for the important life restrictions that this syndrome imposes included of course sport restriction and the consequent psychosocial stigmatization in young people.”

Chest wall deformity was present in the majority of cases (102 patients). Some authors [9, 16–18] observed that pectus deformity may further compromise respiratory function. However, in our research, no patient with pectus excavatum had any respiratory dysfunction.

In accordance with other authors [7, 8], we often observed protrusio acetabuli in patients with Marfan syndrome. According to some authors, patients affected by acetabular protrusion [19] are asymptomatic until hip osteoarthritis develops. Our research supports others [9, 20], because all patients with protrusio acetabuli had mild or moderate hip pain and restricted range of motion. Severe arthritis of the hip was present in only three cases. However, in our research, only 25% of patients were above 40 years of age and so we cannot rule out the notion that osteoarthritis of the hip can develop with age.

Dural ectasia has been observed in 56%–65% of patients with Marfan syndrome [11]. We discovered dural ectasia in only 3 patients (2%). However, we evaluated MRI of the lumbosacral spine only in the few cases who had practiced this exam independently since CT or MRI of the lumbosacral spine was not included in our protocol. The MRI of the lumbosacral spine is currently more and more often used to detect dural ectasia, representing one of the major diagnostic criteria for Marfan syndrome [3, 4].

Scoliosis is a frequent manifestation of Marfan syndrome and may progress rapidly during growth spurts leading to marked deformity [21]. Joseph et al. [20] in a series of patients with definite Marfan syndrome reported an incidence of scoliosis of 100%. Many authors, however, report that scoliosis affects about 60% of patients with Marfan syndrome [21, 22] although in many cases the severity of the scoliotic curves is not reported. We observed scoliosis in about 25% of cases but we considered only curves > 20° measured by Cobb method.

Skeletal abnormalities are fundamental for the diagnosis of this syndrome, although they do not cause sudden or premature death. Musculoskeletal tissues are some of the most obviously involved tissues in Marfan syndrome but these manifestations are often age dependent. Some musculoskeletal abnormalities, in fact, are absent or less evident during the skeletal growth and we agree with Dean [5] and with Coron et al. [23] that the Ghent criteria are unreliable for children. In fact, as described in the revised Ghent criteria [4], clinical decision can be difficult in children.

In 2013, Mueller et al. [24] published a diagnostic tool for risk stratification of suspected paediatric patients with Marfan syndrome. However, this kid-short Marfan score (Kid-SMS9), as commented on in the authors' conclusion, is “an additional tool for general paediatricians and paediatric cardiologists without expansive or age limited investigation.”

In our research, Marfan syndrome was initially diagnosed in only 28 patients (19%) under 14 years of age; this data confirmed that the diagnosis of Marfan syndrome is difficult in children.

Musculoskeletal anomalies were observed in all 146 patients with Marfan syndrome. The more frequent encountered abnormalities were associated wrist and thumb sign (60.2%); pectus carinatum deformity (39.7%); pectus excavatum or chest asymmetry (30.1%); flatfoot (33.5%), and reduced upper segment to lower segment (US/LS) ratio or increased arm span-to-height ratio (36.9%).

The patients surgically treated for aortic complications were affected by skeletal deformities. Such deformities, however, were present also in patients who did not undergo cardiac surgery and we did not observe a secure statistical correlation between the extent of orthopaedic involvement and aortic complications.

In the 28 children examined, the associated “wrist and thumb sign” represented the more frequent skeletal abnormality, similar to adults. On the contrary, we observed a greater frequency of hindfoot deformity (46.4%) and of severe flatfoot (60.7%) compared to the same deformities observed in patients at the end of skeletal growth.

Although the presence of single skeletal features is less important than the combination of skeletal features for the diagnosis of Marfan syndrome, based on our research, the aforementioned musculoskeletal deformities are very significant for the diagnosis of Marfan syndrome.

We could speculate that the clinical signs in the upper and lower limb extremities, above all hands and feet, appear first in Marfan syndrome because the rate of growth of limbs is not constant and varies with age. In fact, growth is much quicker during early childhood and, in particular, it is much more rapid in the distal segments than in the proximal bones. On the contrary, the growth plates are influenced by the action of sex hormones that slow down the growth of long bones, while they stimulate vertebral cartilage and pelvis, so that the arrest of the growth of the limbs corresponds to considerable increased growth of the trunk that continues for a few years after the pubertal crisis [25–27]. In conclusion, in agreement with the current literature, our results seem to confirm that the diagnosis of MFS is difficult in children or in teenagers, but the association with family history and ocular findings is very helpful for the diagnosis of this syndrome. Moreover, according to our data, a good examination of the limbs is very important for an early diagnosis of Marfan syndrome in young patients because the typical deformities of the extremities are often the only obvious orthopaedic clinical signs. Chest and spine deformities are less frequent during childhood but they become more evident towards the end of adolescence and adulthood.

## References

[1] Dietz H. C., Cutting G. R., Pyeritz R. E. (1991). Marfan syndrome caused by a recurrent de novo missense mutation in the fibrillin gene. *Nature*.

[2] Pyeritz R. E., McKusick V. A. (1979). The Marfan syndrome: diagnosis and management. *The New England Journal of Medicine*.

[3] De Paepe A., Devereux R. B., Dietz H. C., Hennekam R. C. M., Pyeritz R. E. (1996). Revised diagnostic criteria for the Marfan syndrome. *American Journal of Medical Genetics*.

[4] Loeys B. L., Dietz H. C., Braverman A. C. (2010). The revised Ghent nosology for the Marfan syndrome. *Journal of Medical Genetics*.

[5] Dean J. C. S. (2007). Marfan syndrome: clinical diagnosis and management. *European Journal of Human Genetics*.

[6] Marfan A. B. (1896). Un cas de deformation congenitale des quatre members plus prononcee aux extremites characterise per l'allongment des os avec un certain degree d’amincissement. *Bulletins et Mémoires de la Société Médicale des Hôpitaux de Paris*.

[7] Van De Velde S., Fillman R., Yandow S. (2006). The aetiology of protrusio acetabuli: literature review from 1824 to 2006. *Acta Orthopaedica Belgica*.

[8] Do T., Giampietro P. F., Burke S. W. (2000). The incidence of protrusio acetabuli in Marfan's syndrome and its relationship to bone mineral density. *Journal of Pediatric Orthopaedics*.

[9] Hohle B. (1978). Familiar occurrence of protusio acetabuli. *Beiträge zur Orthopädie und Traumatologie*.

[10] Pyeritz R. E., Fishman E. K., Bernhardt B. A., Siegelman S. S. (1988). Dural ectasia is a common feature of the Marfan syndrome. *American Journal of Human Genetics*.

[11] Fattori R., Nienaber C. A., Descovich B. (1999). Importance of dural ectasia in phenotypic assessment of Marfan's syndrome. *The Lancet*.

[12] Ahn N. U., Sponseller P. D., Ahn U. M. (2000). Dural ectasia in the Marfan syndrome: MR and CT findings and criteria. *Genetics in Medicine*.

[13] Ha H. I., Seo F. B., Lee S. H. (2007). Imaging of Marfan syndrome: multysystemic manifestations. *RadioGraphics*.

[14] Walker B. A., Murdoch J. L. (1970). The wrist sign. A useful physical finding in the Marfan syndrome. *Archives of Internal Medicine*.

[15] Steinberg I. (1966). A simple screening test for the Marfan syndrome. *The American Journal of Roentgenology, Radium Therapy, and Nuclear Medicine*.

[16] Streeten E. A., Murphy E. A., Pyeritz R. E. (1987). Pulmonary function in the Marfan syndrome. *Chest*.

[17] Scherer L. R., Arn P. H., Dressel D. A., Pyeritz R. M., Haller J. A. (1988). Surgical management of children and young adults with marfan syndrome and pectus excavatum. *Journal of Pediatric Surgery*.

[18] Williams A. M., Crabbe D. C. G. (2003). Pectus deformities of the anterior chest wall. *Paediatric Respiratory Reviews*.

[19] Jones K. B., Sponseller P. D., Erkula G. (2007). Symposium on the musculoskeletal aspects of marfan syndrome: meeting report and state of the science. *Journal of Orthopaedic Research*.

[20] Joseph K. N., Kane H. A., Milner R. S., Steg N. L., Williamson M. B., Bowen J. R. (1992). Orthopedic aspects of the marfan phenotype. *Clinical Orthopaedics and Related Research*.

[21] Pyeritz R. E., Francke U. (1993). The Second International Symposium on the Marfan syndrome. *American Journal of Medical Genetics*.

[22] Sponseller P. D., Hobbs W., Riley L. H., Pyeritz R. E. (1995). The thoracolumbar spine in Marfan syndrome. *The Journal of Bone & Joint Surgery—American Volume*.

[23] Coron F., Rousseau T., Jondeau G. (2012). What do French patients and geneticists think about prenatal and preimplantation diagnoses in Marfan syndrome?. *Prenatal Diagnosis*.

[24] Mueller G. C., Stark V., Steiner K., Weil J., von Kodolitsch Y., Mir T. S. (2013). The Kid-Short Marfan Score (Kid-SMS)—an easy executable risk score for suspected paediatric patients with Marfan syndrome. *Acta Paediatrica*.

[25] Ippolito E., Postacchini F., Scola E. (1983). Skeletal growth in normal and pathological conditions. *Italian Journal of Orthopaedics and Traumatology*.

[26] Ogden J. A. (1982). Skeletal growth mechanism injury patterns. *Journal of Pediatric Orthopaedics*.

[27] Tanner J. M. (1973). Growing up. *Scientific American*.

